# Invasive ductal carcinoma of the breast with osteoclast-like giant cells and clear cell features: a case report of a novel finding and review of the literature

**DOI:** 10.1186/s12957-016-0982-6

**Published:** 2016-08-26

**Authors:** Nicole K. Zagelbaum, Michael F. Ward, Nader Okby, Howard Karpoff

**Affiliations:** 1 Touro College of Osteopathic Medicine, 230 W 125th St #1, New York, NY 10027 USA; 2 Orange Regional Medical Center, 707 East Main Street, Middletown, NY 10940 USA

**Keywords:** Carcinoma, Breast, Osteoclast-like giant cells, Clear cells

## Abstract

**Background:**

Osteoclast-like giant cells (OLGCs) are a rare histologic finding within a tumor of the breast. Although there has been discussion as to the pathogenesis and prognosis related to this finding, our understanding of its significance remains inconclusive. Clear cells are another unique histologic finding in breast tumors and are typically associated with tumors arising in other organs such as renal cell carcinoma.

**Case presentation:**

This is a case report of a 64-year-old female who presented with one tumor identified as invasive ductal carcinoma with a combination of OLGCs and clear cell features.

**Conclusions:**

To our knowledge, this combination of findings has not been previously described in the literature and therefore represents another morphologic manifestation of breast carcinoma. As patients are diagnosed earlier and live longer, a growing number of these rare variants may be recognized and provide opportunities to further our understanding of the associated molecular pathways which could contribute to the possibility of therapeutic intervention.

## Background

Breast cancer is the most commonly diagnosed noncutaneous cancer and the second leading cause of cancer death among women worldwide [1]. In the USA, the incidence of breast cancer in women increased from 105.1 per 100,000 in 1975 to 129.6 per 100,000 in 2012 [2, 3]. Simultaneously, the mortality has decreased by 30 % since the 1990s resulting in a prevalence of over 3.1 million diagnosed breast cancer cases in the USA as of 2014 [1, 4].

Breast cancer progression is a complex and multifaceted subject. Prognosis is based on a combination of factors including lymph node status, tumor size, and histology, as well as expression of hormone and growth receptors [5–7]. Histologic reports and proteomic analysis have determined that most breast malignancies arise from epithelial tissue and that ductal and lobular carcinomas make up 75 and 15 % of invasive cancers, respectively [2, 8, 9]. Several rarer subtypes including mucinous, clear cell, OLGCs, and pleomorphic carcinomas account for the remaining 10 % of all cases and continue to be relatively unexplored due to few reported cases and a lack of large statistically significant studies [10]. As the prevalence of breast cancer increases, there should be a simultaneous escalation in the number of these historically rare variants and the need to classify them appropriately as molecular pathways of varying cancers may have important implications on prognosis and treatment.

### Introduction to osteoclast-like giant cells

OLGCs are large multinucleated cells that resemble the morphology and function of histiocytic osteoclasts found in bone [11]. They have typically been associated with several cancers including gallbladder, liver, and thyroid [12–14]. Agnatis first reported OLGCs as a component of a primary breast malignancy in 1979 [15]. They are found in only 0.5–1.2 % of all primary breast carcinomas and to date approximately 200 cases of OLGCs associated with breast malignancy have been reported [16, 17]. OLGCs have been detected mostly in association with invasive metaplastic carcinoma but may be seen with other histologic variants including lobular, tubular, mucinous, and papillary patterns [10, 16].

### Introduction to clear cells

Clear cells are recognized by histologic findings that result from the removal of cytoplasmic inclusions during tissue processing. Various cellular components may result in a clear appearance and histochemical staining can be used to determine the contents of the cell, although it is not routinely performed. Some common contents include lipid, mucin, or glycogen [18, 19]. Clear cells are traditionally found in carcinomas of the kidney, ovary, vagina, cervix, endometrium, and salivary glands [20–22]. Rarely, clear cells have also been identified in several types of breast carcinomas including ductal, lobular, adenocarcinoma, squamous cell carcinomas, and metastases from other organs [23, 24]. Hull first described the presence of glycogen-rich clear cells as a separate histologic category of invasive ductal carcinoma of the breast in 1981 [18]. Fewer than 150 cases have been reported in the literature as of 2014 [25].

This is a case report of a patient who presents with a previously undescribed combination of these two unique histologic categories of invasive ductal carcinoma. We also provide a review of the literature on these rare characteristics of breast carcinoma that have been previously reported in separate studies.

## Case presentation

**Fig. 1:**
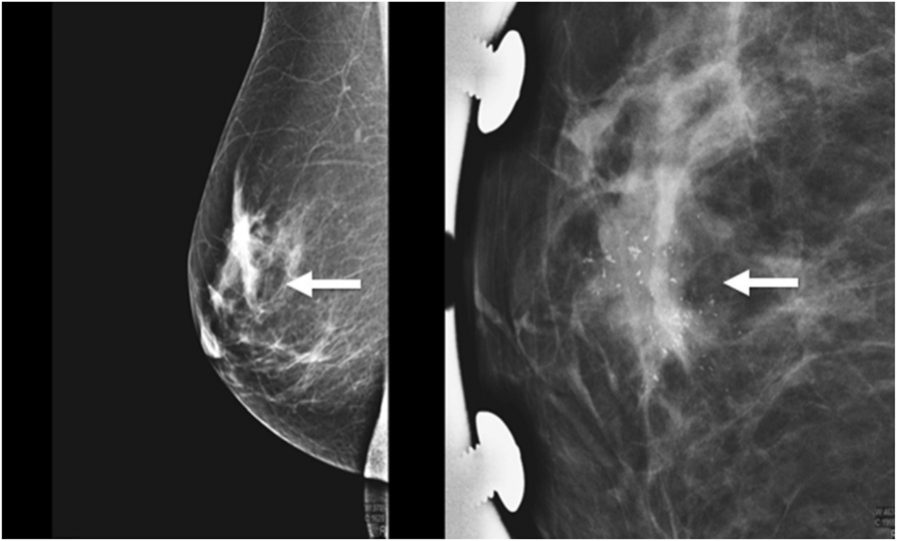
Initial MLO view mammography demonstrating an irregularly bordered mass (*left*, *arrow*). Magnified view of the right breast showing several pleomorphic microcalcifications (*right*, *arrow*) contained within the mass

**Fig. 2:**
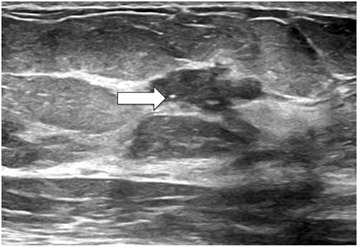
Ultrasound image of right breast mass where several small calcifications can be seen (*arrow*), representing an uncommon sonographic finding

A 64-year-old Caucasian female with no personal or family history of breast or ovarian cancer presented for routine screening mammography. Imaging showed an irregular 4-cm mass in the upper outer quadrant of the right breast containing several pleomorphic calcifications (Fig. 1). This lesion was assigned a Breast Imaging Reporting and Data System (BIRADS) score of 4, representing a suspicious abnormality where biopsy is recommended [26]. Ultrasound (US) identified a mass with angular margins, calcifications, and hypervascularity suspicious for invasive ductal carcinoma (Fig. 2). The lesion was sampled using vacuum-assisted US-guided biopsy with a 14-gauge needle, and the biopsy was placed in 10 % neutral buffered formalin and forwarded to pathology for processing.

**Fig. 3:**
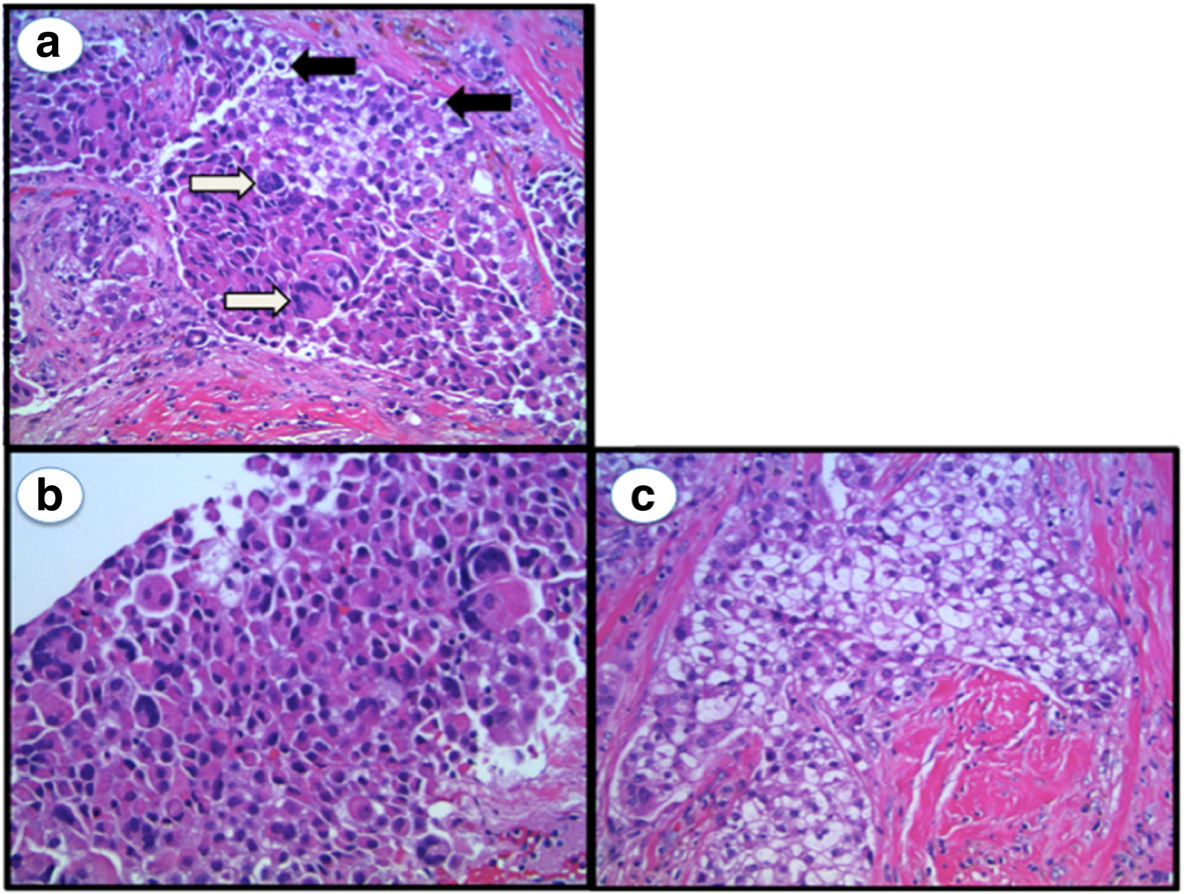
H&E stain demonstrating invasive ductal carcinoma. **a** Both OLGCs (*white arrows*) and clear cells (*black arrows*) are present throughout the tumor. **b** Large focus of OLGCs. **c** Predominant clear cell features

**Fig. 4:**
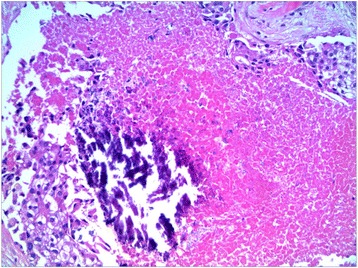
H&E stain demonstrating central necrosis and associated calcifications

Grossly, the biopsy consisted of four red yellow cylindrical fibrofatty soft tissue cores ranging from 1.5 to 1.7 cm in length. Hematoxylin and eosin (H&E) sections were microscopically examined and demonstrated invasive nests of cuboidal cells with ample amphiphilic cytoplasm. In addition, large multinucleated cells with pink cytoplasm, intracellular granular inclusions, and increased nuclear to cytoplasmic ratio were identified. Small polygonal cells with centrally located nuclei and clear cytoplasm were noted as well as areas of central necrosis and associated calcifications (Figs. 3 and 4).

**Fig. 5:**
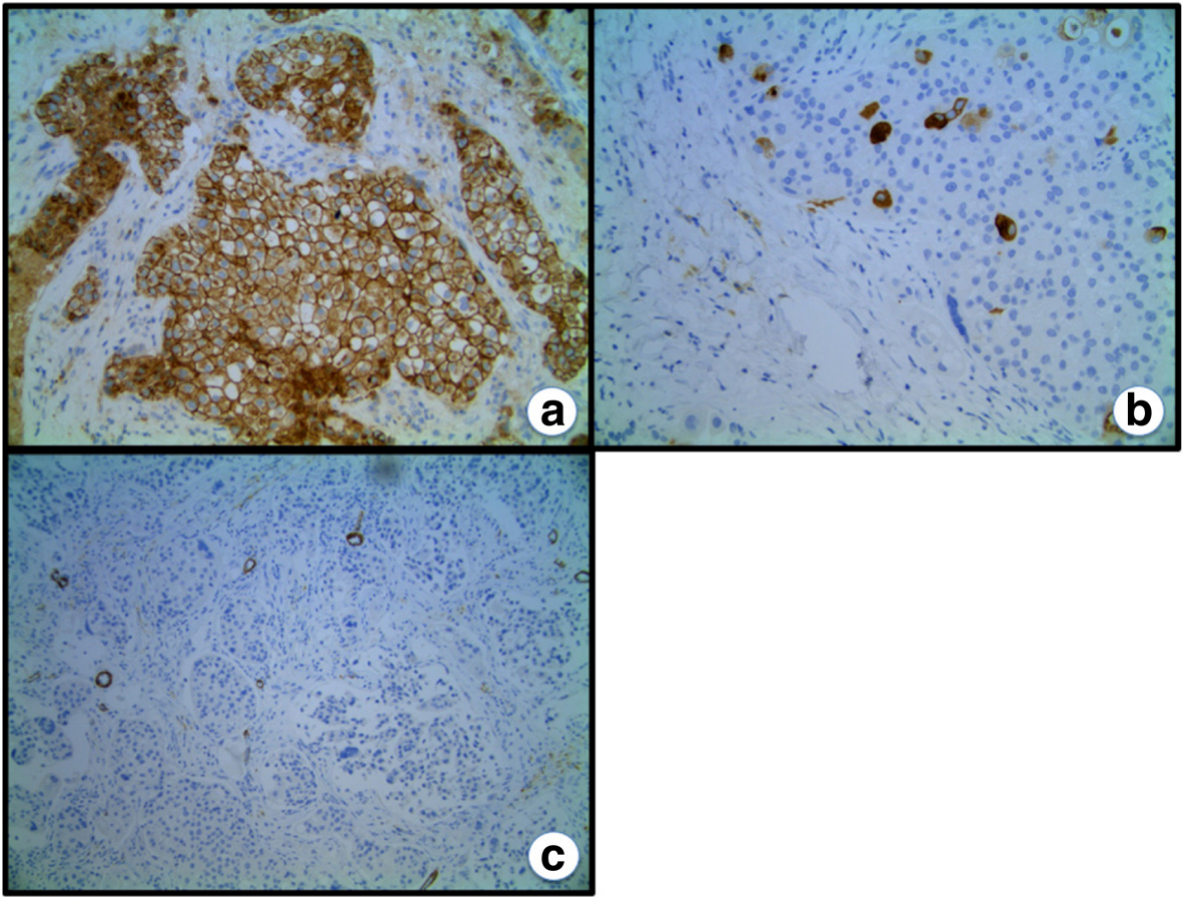
**a** Stain demonstrating positive for GATA3. **b** Focally positive mammaglobin stain, confirming the tumor to be breast in origin. **c** Stain for smooth muscle myosin heavy chain only present in arteriole walls, demonstrating neovascular changes in tumor

Immunohistochemical staining demonstrated tumor cells to be positive GATA3 (Fig. 5a), confirming the lesion to be ductal cell in origin. In addition, mammaglobin was focally positive (Fig. 5b) indicating the tumor to be breast tissue and not a metastasis from another site. Smooth muscle myosin heavy chain was negative, verifying the tumor architecture to be abnormal and invasive (Fig. 5c). These overall findings were consistent with invasive ductal carcinoma with OLGCs and clear cell features. This diagnosis was corroborated by an outside, fellowship-trained breast pathologist. Further immunohistochemical staining found the sample to be positive for estrogen and progesterone receptors and negative for Human Epidermal Growth Factor Receptor 2 (HER2).

### Discussion and review of the literature

#### Osteoclast-like giant cells

OLGCs in association with breast tumors are believed to represent a fusion of several cells of monocyte lineage located in the stroma. The significance of this finding is inconclusive. The 5-year survival rate is about 70 % versus an average overall survival rate of 72 % for similarly staged breast carcinomas [3, 10]. In six cases of invasive carcinomas with OLGCs, Holland did not find an exceptionally different clinical course when compared to typical invasive carcinomas [27]. Agnantis described eight patients with similar results in terms of prognosis and outcome [15]. Other investigations have shown that the average size of an OLGC-containing breast carcinoma is 3 cm and that over one third of patients have axillary metastasis [27]. Cai reviewed 42 cases of OLGC in breast carcinoma and found a majority had a relationship to marked angiogenesis and that this finding portended a poorer prognosis [27, 28].

Much debate and speculation has gone into the origin of OLGCs and their relationship to breast cancer [15, 29]. Markopoulos hypothesized that chemotactic agents produced by the tumor may recruit histiocytes to the region, resulting in this unique histological subtype of breast carcinoma [30]. Interestingly, one study found that OLGCs isolated from an invasive breast cancer were able to digest bone directly in vitro. These were the first cells observed to resorb bone that were not directly harvested from osseous tissue. Unlike osteoclasts, which require the presence of osteoblasts to be stimulated, these OLGCs were directly activated by the presence of parathyroid hormone. Additionally, the cells were not inhibited by calcitonin, demonstrating another key distinction between OLGCs and osteoclasts [11]. These differences provide important clues into the origin of these OLGCs, and more research may be warranted to clarify the significance of these cells.

#### Breast carcinoma with clear cell features

Clear cells are a rare histologic finding in a primary breast cancer and can be seen in several tumor types. Variants reported within primary breast tumors include glycogen-rich clear cell carcinoma (GRCCC), signet-ring, lipid-filled, and secretory carcinomas. Of these, GRCCC is the most common clear cell variant in breast cancer [30]. The current diagnostic criterion for a GRCCC is debatable. One early study defined GRCCC tumors as containing greater than 50 % clear cells [31]. However, the World Health Organization (WHO) definition is a tumor in which greater than 90 % of the neoplastic cells contain clear cytoplasm filled with glycogen [10], reflecting the variability of cell composition seen in breast tumors.

There is conflicting evidence regarding the survival rate of patients diagnosed with GRCCC. Some research suggests a poor prognosis. One case series found that five of its six cases had axillary lymph node involvement at the time of diagnosis and that all five of these patients succumbed to the disease within 7 years [31]. By comparison, the overall 5-year survival rate of all types of breast cancer was 89.4 % between 2005 and 2011 [32]. WHO identifies GRCCC to have a more aggressive course with axillary involvement than other ductal carcinoma variants. However, they acknowledge that prevalence is not yet sufficient to establish large multimodal studies on these relationships [10]. In contrast, Hayes matched GRCCC to other types of invasive breast carcinoma by tumor stage and grade and demonstrated no difference in outcomes [33]. Overall, the consensus is that there have not been enough reported cases to draw significant conclusions on GRCCC’s effect on patient outcomes warranting further investigation on the subject.

The research in the clinical progression of GRCCC is also conflicted. A few case studies suggest low rates of recurrence following tumor excision. Hull presents a case where a patient had no axillary lymph node involved which contained any evidence of neoplasm after mastectomy [18]. Sorensen and Paulsen describe a patient without recurrence or metastasis after a follow-up period of 6 months [34]. Shirley outlines a case where no evidence of metastatic disease was found after 18 months of follow-up [35]. However, Kuroda identifies a propensity for GRCCC to metastasize in a study that aggregated over 700 cases of breast carcinoma in which 20 cases were GRCCC. In these cases, tumor size was an average of 2.6 cm and 35 % of patients had positive lymph nodes in the axillary region [36].

Other clear cell variants tend to have a more insidious progression. Signet-ring cell carcinoma of the breast contains primarily mucinous inclusions and has a 5-year survival rate of 45–60 % [37]. Lipid-rich carcinoma of the breast also has an aggressive course and poor prognosis, with a 33 % 5-year survival rate [38]. Secretory cell carcinoma of the breast has axillary lymph node metastasis in 15–30 % of all cases [19]. Overall, studies have indicated an incomplete understanding of the pathogenesis and prognosis associated with clear cell features in invasive ductal carcinoma of the breast.

Additional case reports imply that underreporting as well as misdiagnosis may be prevalent. Ovanez suggests clear cell carcinoma may mimic the appearance of pseudo-lactating changes in a premenopausal female or reflect benign changes of the breast at any age [25]. Markopoulos reported a case of a woman whose mammogram revealed a 3.5-cm lobular mass which was originally misdiagnosed to be a fibroadenoma but was finally diagnosed as a clear cell carcinoma 4 years later [30]. Aboumrad identified an example where clear cells may be confused with lipid-filled macrophages in fat necrosis of the breast [39]. Metastatic clear cell carcinomas originating in other origins such as the kidney can also mimic clear cell features found in breast carcinoma [23].

## Conclusions

In summary, this paper outlines our current understanding of two rare variants of breast carcinoma and provides a case study involving a unique histologic finding that has not been previously reported. The significance of cytology in the clinical progression of rare tumors of the breast is incompletely understood. The literature to date suggests that certain cell types of breast cancer may correlate with a poorer prognosis. As patients are diagnosed earlier and live longer, a growing number of these rare variants may be recognized and provide opportunities to further our understanding of the associated molecular pathways which could contribute to the possibility of therapeutic intervention. We believe it is important for health practitioners to be aware of these rare tumors as they may impact the development of optimal treatment plans in the future.

## Abbreviations

OLGCs: Osteoclast-like giant cells
BIRADS: Breast Imaging Reporting and Data System
US: Ultrasound
H&E: Hematoxylin and eosin
HER2: Human Epidermal Growth Factor Receptor 2
GRCCC: Glycogen-rich clear cell carcinoma
WHO: World Health Organization
